# Comment on Umemoto et al. Management of Migraine-Associated Vestibulocochlear Disorders. *Audiol. Res.* 2023, *13*, 528–545

**DOI:** 10.3390/audiolres14010015

**Published:** 2024-02-07

**Authors:** Daphne J. Theodorou, Stavroula J. Theodorou, Vasilios Mitsios

**Affiliations:** 1 Department of Radiology, General Hospital of Ioannina, 45444 Ioannina, Greece; 2 Department of Radiology, University Hospital of Ioannina, 45500 Ioannina, Greece; 3 Department of Neurotology and Otolaryngology, General Hospital of Ioannina, 45444 Ioannina, Greece

We read with great interest the recent article by Umemoto, K.K. et al. [1] reviewing migraine, a chronic highly complex and non-curable neurological disorder that comprises an important public health problem. These authors were correct to address the association of migraine with disturbing symptoms and signs related to the inner ear: dizziness, tinnitus, hearing loss, spatial disorientation, and vertigo. Because of accumulating evidence of a shared pathophysiology between migraine and various vestibulocochlear disorders (i.e., positional vertigo, Ménière’s disease), the authors also thoroughly reviewed this association, and developed a useful clinical algorithm for treating symptomatic patients [2,3].

We definitely agree with the authors that patients with migraine-related symptoms are usually clinically underdiagnosed and perhaps receive insufficient treatment. As rapid advances in MR imaging technologies continue to revolutionize radiology, we have used MR imaging to investigate the source of symptoms regarding the inner ear in affected patients. In so doing, in our clinical practice, we have advocated a simple MR imaging protocol of the inner ear that would employ the 3D-constructive interference in steady state (3D-CISS) sequence (or equivalent fast gradient-echo pulse sequence). The CISS sequence is readily available in most high-strength (1.5 T or above) MR units and allows for the fast acquisition of high-resolution images and multiplanar reconstructions in the inner ear. Because of a direct cisternographic effect that enables a depiction of the vestibulocochlear lymph space, the visualization of the fine, minute anatomic structures in the membranous labyrinth and a depiction of the acoustic nerves is feasible [4], sparing the off-label administration of high-dose gadolinium compounds that may be toxic, invasive, time-consuming, expensive, or require high-strength MR units [5] (Figure 1). In our institution, this simple approach of imaging the peripheral vestibular system complements routine brain MR imaging studies in migraine sufferers. Standard brain screen MR imaging protocols allow, foremost, for a general assessment of the cerebral hemispheres and cortex, the cerebellum, the brainstem and trigeminal nerves, and the thalami, anatomic areas that play a key role in the pathogenesis of migraine [2,3,6,7]. As such, a fast MR imaging survey of the brain and inner ear may need to be pursued in migraine patients, with or without overt otologic problems.

Migraine can be chronic, and repeated episodes may cause severe impairment of daily activities in affected individuals. MR imaging can detect changes in the inner ear and brain and is useful in the diagnosis of various abnormal conditions associated with migraine. In any case, the authors would agree that MR imaging is a powerful tool that needs to be added to the diagnostic armamentarium of clinicians managing migraine and its debilitating neurological symptoms.

## Figures and Tables

**Figure 1:**
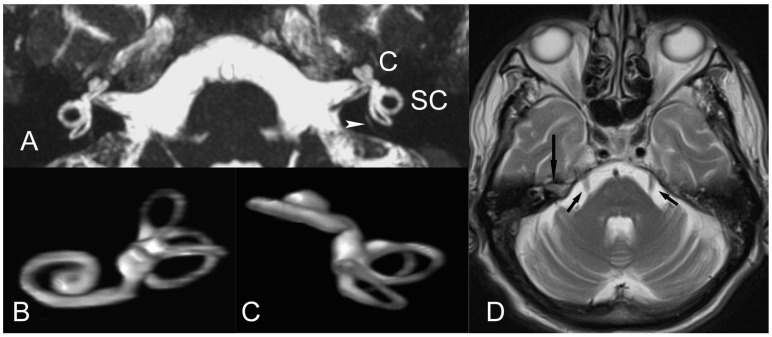
(**A**) Axial non-gadolinium-enhanced 3D-CISS image (volumetric technique) delineates normal anatomy of the inner ear and the membranous labyrinthine structures on both sides, in a 16-year-old patient with migraine headaches, vertigo, and tinnitus. At this image level, a portion of the fine vestibular aqueduct (arrowhead) is seen. C, cochlea; SC, semicircular canals. (**B**,**C**) Three-dimensional-volume rendering (VR) image reconstructions of (A) depict normal, membranous labyrinth. (**D**) Axial T2-weighted MR image delineates trigeminal nerves (V) (arrows). The vestibulocochlear (auditory) nerve (VIII) also is seen on the right (long arrow).
